# Erythro-myeloid progenitors can differentiate from endothelial cells and modulate embryonic vascular remodeling

**DOI:** 10.1038/srep43817

**Published:** 2017-03-08

**Authors:** Bahar Kasaai, Vincenza Caolo, Hanna M. Peacock, Stephanie Lehoux, Elisa Gomez-Perdiguero, Aernout Luttun, Elizabeth A. V. Jones

**Affiliations:** 1 Department of Cardiovascular Sciences, Centre for Molecular and Vascular Biology, KU Leuven, Herestraat 49 - box 911, 3000 Leuven, Belgium; 2 Institute of Human Genetics, CNRS, 141 rue de la Cardonille, 34396, Montpellier, France; 3 Lady Davis Institute, Department of Experimental Medicine, McGill University, 3755 Ch. Côte-Ste-Catherine, Montréal, QC, H3T 1E2, Canada; 4 CNRS URM 3738, Institut Pasteur, 25-28 Rue du Docteur Roux, Paris 75015, France

## Abstract

Erythro-myeloid progenitors (EMPs) were recently described to arise from the yolk sac endothelium, just prior to vascular remodeling, and are the source of adult/post-natal tissue resident macrophages. Questions remain, however, concerning whether EMPs differentiate directly from the endothelium or merely pass through. We provide the first evidence *in vivo* that EMPs can emerge directly from endothelial cells (ECs) and demonstrate a role for these cells in vascular development. We find that EMPs express most EC markers but late EMPs and EMP-derived cells do not take up acetylated low-density lipoprotein (AcLDL), as ECs do. When the endothelium is labelled with AcLDL before EMPs differentiate, EMPs and EMP-derived cells arise that are AcLDL^+^. If AcLDL is injected after the onset of EMP differentiation, however, the majority of EMP-derived cells are not double labelled. We find that cell division precedes entry of EMPs into circulation, and that blood flow facilitates the transition of EMPs from the endothelium into circulation in a nitric oxide-dependent manner. In gain-of-function studies, we inject the CSF1-Fc ligand in embryos and found that this increases the number of CSF1R^+^ cells, which localize to the venous plexus and significantly disrupt venous remodeling. This is the first study to definitively establish that EMPs arise from the endothelium *in vivo* and show a role for early myeloid cells in vascular development.

The first myeloid cells develop in the mouse embryo during yolk sac hematopoiesis from primitive macrophage-restricted progenitors at embryonic day 7.5 (E7.5) and from erythro-myeloid progenitors (EMPs) at E8.5^1^,^2^. Recent fate mapping reports show that EMPs are sufficient to support embryo survival until birth^3^, and can develop into tissue resident macrophages that have self-renewing properties^2^,^4^,^5^,^6^,^7^. While EMPs can differentiate into macrophages within the yolk sac, they also colonize the fetal liver from E9^8^ and differentiate into erythrocytes, megakaryocytes, macrophages, monocytes, granulocytes, and mast cells^2^. Whether EMP-derived macrophages bypass the monocyte stage of development is still controversial as very little is known about their differentiation pathway^7^,^9^. Understanding EMP renewal and differentiation is important, since defects in EMP development could result in long-term effects on tissue resident macrophages.

In mouse embryos, endothelial cells of the aorta-gonad-mesonephros (AGM) region and vitelline arteries have been shown to produce hematopoietic stem cells (HSCs)^10^,^11^,^12^,^13^,^14^,^15^. While surface marker analyses imply that EMPs also derive from endothelial cells^2^,^4^,^16^,^17^, to date this has not been conclusively demonstrated. For instance, we were the first to identify that early EMPs arise from Tie2^+^ cells^2^. Our findings were not intended to assert an endothelial cell origin of EMPs, because Tie2 is not endothelial-specific at this stage but is also expressed by mesodermal cells^18^,^19^. Nevertheless, several groups subsequently showed that EMPs express a number of endothelial cell genes, including VE-Cadherin, PECAM-1, CD105 and CD34^17^,^20^,^21^. Importantly, the expression of common markers between endothelial precursors and hematopoietic cells does not prove a common origin^22^,^23^, particularly in the absence of *in vivo* validation. EMPs have also been observed in the vessel wall^20^,^21^; however, it can be argued that EMPs actually develop in the mesoderm near vessels and only transiently pass through the endothelium, as has been observed for HSCs in the AGM^24^. As such, prior to this report, evidence that EMPs arise directly from endothelial cells was indirect and required substantiation.

EMPs are first observed at E8.5^2^, a stage when blood flow and vascular remodeling initiates. This raises the question as to whether blood flow is required for their development, and whether EMPs play a role in early vascular development. While the function of yolk sac EMPs in early vascular development is elusive, adult macrophages and myeloid cells are known to modulate vascular remodeling by promoting angiogenesis (sprouting of new vessels) and arteriogenesis (changes in vessel diameter in response to flow dynamics)^25^,^26^,^27^. To date, the earliest vascular function attributed to yolk sac-derived macrophages is in mediating vessel anastomosis at E11.5^28^,^29^, but these cells represent tissue-differentiated cells and not EMPs *per se. Csf1r*^−/−^ mice lack yolk sac-derived macrophages at E12.5^5^, yet no pre-natal vascular abnormalities were reported in these mice^30^. The absence of yolk sac derived macrophages was not verified prior to E12.5 and thus it is not clear if the mutation affected their initial differentiation or their long-term survival. More recent studies in *Ncx*^−/−^ mouse embryos, which lack blood flow, found no difference in the number or morphology of EMPs at E10.0^20^, suggesting that unlike for HSCs^31^,^32^, EMP differentiation does not require hemodynamic forces.

To address these points, we investigated the relationships of EMPs with the endothelium, blood flow, and vascular remodeling. Similar to recent reports^20^, we found that EMPs reside in the blood vessels of the yolk sac at E8.5 and enter circulation over the following day. Further to this, we delineated the precise origins of EMPs. We found that the majority of EMPs develop from the blood islands, but that a third of circulating EMPs emerge from the capillary plexus and initially have an elongated and endothelial-like phenotype. At E9.0, EMPs are positive for most endothelial cell markers, with the exception of acetylated low-density lipoprotein (AcLDL), which is taken up by endothelial cells but not EMPs. We therefore investigated earlier stages and find that AcLDL uptake is present in Runx1^+^ cells, but that this ability to uptake AcLDL is lost later. We then use AcLDL labelling combined with *in vivo* time-lapse imaging^33^, to capture for the first time, EMP differentiation from the endothelium of developing mouse embryos. Contrary to previous reports, we found that blood flow facilitates the transition of EMPs into circulation through a nitric oxide-dependent mechanism. Using gain-of-function experiments, we show that EMPs in the yolk sac are required for normal remodeling of the venous plexus. This report identifies novel mechanisms and behavior of EMPs during embryonic vascular remodeling.

## Results

### One third of EMPs arise from blood vessels outside of the blood islands

To genetically label EMPs and EMP-derived cells during early vascular development, we used two transgenic myeloid-specific Cre recombinase mouse models: *Csf1r*^*iCre*^ (constitutive) and *Csf1r*^*MeriCreMer*^ (tamoxifen-inducible). These mice were mated with *Rosa26*^*tdTomato*^ mice to fluorescently label *Csf1r*-expressing cells and their progeny with tdTomato (tdT^+^). For the inducible iCre transgenic model, a single pulse of tamoxifen was administered to the dam at E7.75. We previously showed that this specifically labels EMPs^2^, where 100% of labeled cells at E8.5 (11–13 somite pairs) are c-kit^+^, CD45^low^, AA4.1^+^. In agreement with previous reports^2^,^4^,^5^,^6^, we found that yolk sac EMPs can contribute to tissue resident macrophages in neonate mouse tissues (Supplemental Fig. 1). It was previously reported that early EMPs express *Csf1r* mRNA but not CSF1R protein at E9.0^7^. We therefore also examined the expression of CSF1R protein in tdT^+^ cells. Though not all CSF1R^+^ cells expressed tdT at E8.5 (10 somite pairs), indicating that Cre-mediated excision is not 100% penetrant, all tdT^+^ cells did express CSF1R protein (Supplemental Fig. 2). By E9.5 (25 somite pairs), some tdT^+^ cells no longer expressed CSF1R protein (Supplemental Fig. 2). The discrepancy with previous reports may simply be due to difference in the genetic backgrounds of the mouse strains.

**Figure 1:**
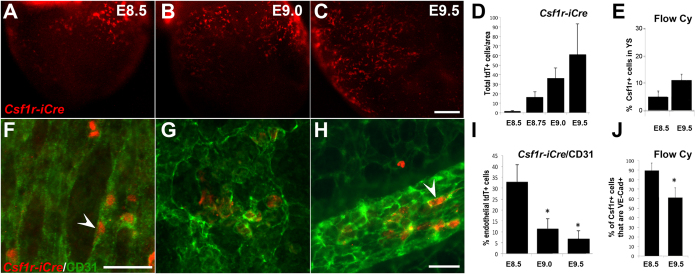
EMPs and EMP-derived cells increase with developmental stage but lose their endothelial cell characteristics at later stages. *Csf1r*^*iCre*^*Rosa26*^*tdT*^ yolk sacs were harvested at E8.5 (10 somite pairs, **A**) E9.0 (15 somite pairs, **B**) and E9.5 (25 somite pairs, **C**) to visualize tdT^+^ cells. The number of total of tdT+ cells increased based on immunofluorescence of *Csf1r*^*iCre*^*Rosa26*^*tdT*^ embryos (**D**, n = 4–5 per stage) and of CSF1R^+^ cells based on flow cytometry of wild type embryos (**E**, n = 6 at E8.5 and n = 9 at E9.5). Blood vessels were counterstained with CD31 (**F–H**, green). Endothelial-like tdT^+^ cells (**F** and **H**, arrowheads) were blindly quantified based on 3 phenotypic criteria (CD31 co-expression, cell shape, and vessel integration) by immunofluoresence. Analysis was limited to yolk sac vessels proximal to the embryo proper. The number of tdT^+^ cells with an endothelial phenotype was initially 32% but decreased in older embryos (**I**, n = 4–5 embryos per stage). The percentage of CSF1R^+^ cells that co-stained for VE-Cadherin was also measured by flow cytometry and confirmed the decrease in endothelial staining in older embryos (J). Scale bars: 250 μm (**A**–**C**), 50 μm. (**F**–**H**) All values are mean ± SEM. *p < 0.05; two-tailed Student’s t-test.

We then investigated the development of yolk sac EMPs using cell lineage tracing and immunofluorescence. As previously shown^2^,^34^, EMPs are first detected at E8.5 (7–10 somite pairs, Fig. 1A), and the number of EMPs and EMP-derived cells increases by E9.0 (Fig. 1B) and further by E9.5 (Fig. 1C). The increase in EMP and EMP-derived cells was quantified by immunofluorescence on *Csf1r*^*iCre*^ *Rosa26*^*tdT*^ embryos (Fig. 1D) and by flow cytometry of CSF1R^+^ cells in wild-type yolk sacs (Fig. 1E, Supplemental Fig. 3 for gating).

Morphologically, both CSF1R^+^ wild-type cells and *Csf1r*^*iCre*^ *Rosa26*^*tdT*^ labeled cells displayed two distinct phenotypes: the majority had a round or ovoid myeloid shape, while a subset adopted a flattened endothelial shape and were embedded within the blood vessel intima (Fig. 1F–H arrowheads). Blood vessels of the yolk sac were visualized with CD31 staining. We quantified the number of tdT^+^ cells with an endothelial phenotype based on three stringent criteria: CD31^+^ endothelial expression, flat and elongated morphology typical of endothelial cells, and integration in the vessel wall (Fig. 1I). We found that at the onset of circulation at E8.5, 32.8 ± 8.0%% of tdT^+^ cells had an endothelial phenotype (Fig. 1I). Thereafter, the percentage of endothelial-like cells decreased to 11.6 ± 4.5% at E9.0 and 6.8 ± 3.8%% at E9.5. To investigate whether this coincided with loss of endothelial cell gene expression, we quantified the percentage of CSF1R^+^ cells that also stained positive for VE-Cadherin in wild type yolk sacs by flow cytometry (Fig. 1J). We found that at E8.5, 89.4 ± 7.6%% of CSF1R^+^ cells are also positive for VE-Cadherin and that this was reduced to 60.8 ± 10.3% at E9.5. Both analyses confirm a decrease of endothelial characteristics for EMPs and EMP-derived cells with developmental stage; however, the results also indicate that CSF1R^+^ cells outside the endothelium continue to express endothelial cell markers. We further confirmed the endothelial association of EMPs using the endothelial reporter *Cdh5*(Pac) ^CreERT2^ mouse^35^. Dams were tamoxifen-induced when embryos were E7.5, yolk sacs were harvested at E9.0 and immuno-stained for CSF1R (Supplemental Fig. 4). We found that a high number of *Cdh5*-derived cells (Supplemental Fig. 4, red vessels) co-express CSF1R^+^ (Supplemental Fig. 4B and C, white arrowheads). However, not all CSF1R^+^ cells (green) were *Cdh5*-derived (red), which suggests that either only a subset of CSF1R^+^ cells arise from *Cdh5*-expressing endothelial cells or, alternatively, that Cre excision was not 100% penetrant.

**Figure 2:**
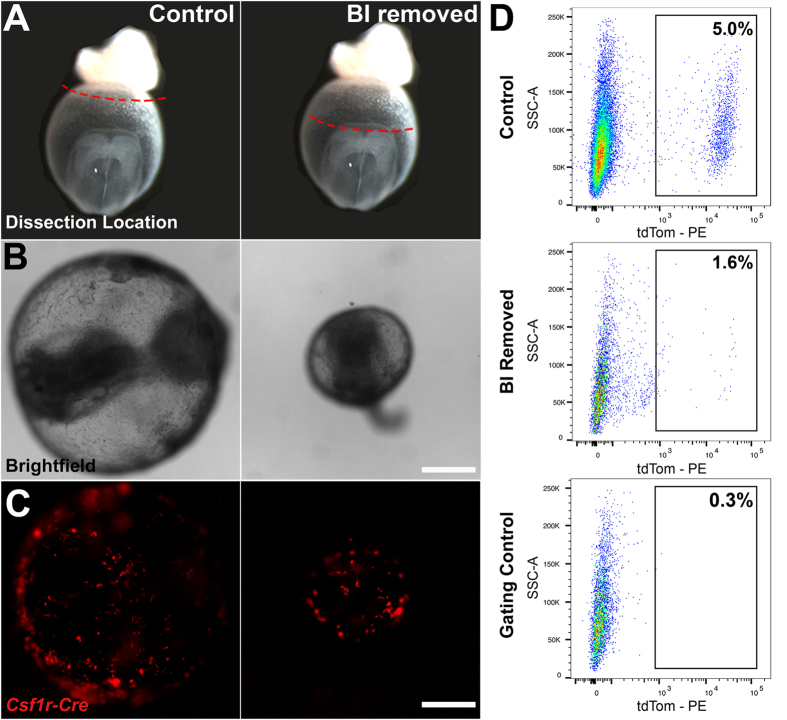
EMPs develop from hematopoietic blood islands and the vascular plexus. Schematic of embryos dissected at E8.0 (3–5 somite pairs) and cultured with only the ectoplacental cone removed (control) or with the blood islands (BI) excised (**A**). Tissues above red dotted line were removed. Brightfield images after culture demonstrate that embryos develop well and that the yolk sac is inflated in both control and experimental embryos (**B**, n = 5 for each condition). Fluorescent images of *Csf1r*^*iCre*^*Rosa26*^*tdT*^ control and blood island-depleted embryos show tdT^+^ cells (red) develop in the absence of BI but in reduced numbers (**C**). Flow cytometry analysis was used to quantify differences in the percentage of tdT^+^ yolk sac cells between control and blood island depleted embryos (**D**; n = 4 control, SEM = 0.8; n = 3 BI removed, SEM = 0.6, p = 0.01). Scale bars: 500 μm (**B**–**C**). Two-tailed Student’s t-test.

We observed a differential location of yolk sac EMPs and EMP-derived cells at E8.5 based on phenotype: most of the rounded cells were located in the distal hematopoietic blood islands, whereas those with an endothelial morphology were located in regions of the yolk sac proximal to the embryo (Supplemental Fig. 5). We therefore cultured embryos with and without the blood islands to investigate the different sites of origin. *Csf1r*^*iCre*^*Rosa26*^*tdT*^ embryos were harvested at E8.25 (3–5 somite pairs) prior to EMP development and the onset of flow. As a sham experiment, only the ectoplacental cone was removed, whereas in experimental embryos, both the ectoplacental cone and blood islands were removed (Fig. 2A). Embryos were cultured for 16 hours. At the end of culture, yolk sacs had healed and were inflated, even in embryos where the blood islands were removed (Fig. 2B). All embryos had viable heart beats. The morphology of the embryo proper was normal, but somewhat delayed in both sham and experimental groups such that neither set of embryos turned. We assessed the efficiency of blood island removal by quantifying erythroblasts by flow cytometry, and found a 96% reduction in Ter119^+^ erythroblast post-operation. Specifically, an average of 5600 ± 530 Ter119^+^ cells were present per sham-operated embryo (n = 4), compared to 210 ± 37 Ter119^+^ cells in blood-island removed embryos (n = 5). Since a hemogenic endothelium is present at this stage, complete ablation of all erythroblasts would not occur even with removal of the blood islands. We also observed that tdT^+^ cells developed in both control and experimental embryos (Fig. 2C), and quantified them by flow cytometry (Fig. 2D). We found that in control embryos, 5.0 ± 0.8% of cells were tdT^+^, compared to only 1.6 ± 0.6% in embryos cultured without blood islands. These culture experiments confirm that 32% of EMPs (i.e., 1.6% divided by 5.0%) develop in the yolk sac outside the blood islands, which corroborates our results obtained using endothelial cell morphology as criteria (Fig. 1I).

### EMPs only take up AcLDL while in the vessel wall

**Figure 3:**
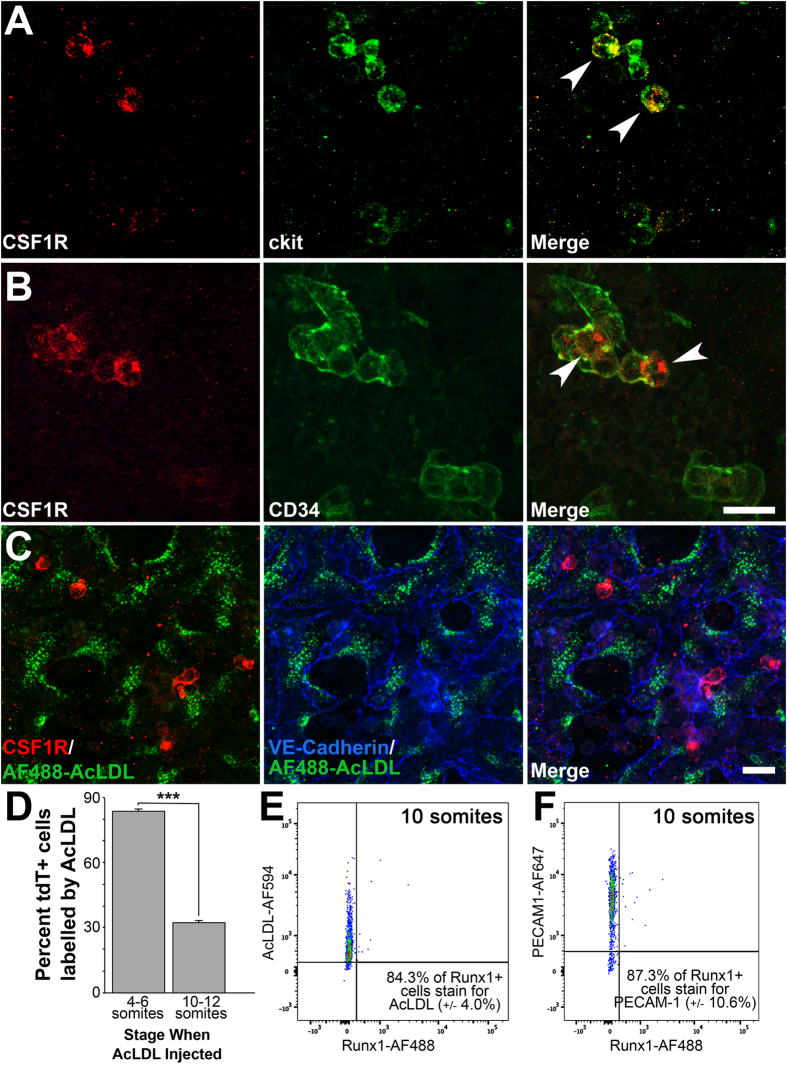
CSF1R^+^ EMPs are positive for several hematopoietic/endothelial cell markers but do not take up AcLDL. Confocal microscopy images of wild-type yolk sacs dissected at E9.0 (15 somite pairs) and immunostained for CSF1R and for hemogenic markers c-kit (**A**, n = 3) and CD34 (**B**, n = 3). White arrowheads indicate CSF1R^+^ cells that co-express hemogenic/hematopoietic CD34 and c-kit. Though CSF1R^+^ cells expressed most endothelial cell markers, they did not take up AcLDL as endothelial cells do (**C**, green). Counterstaining with VE-Cadherin shows AcLDL^+^ endothelial cells (blue). Embryos were then dissected at either E8.0 (4–6 somite pairs) or E8.5 (10–12 somite pairs), injected with AF488-AcLDL and cultured for 24 hours. Quantification of tdT^+^ AcLDL^+^ cells show that the majority of tdT^+^ cells are also AcLDL^+^ when the injection is done before EMPs arise, however only a third of tdT^+^ cells are labelled if injected after EMPs arise (**D**). Embryos at E8.5 (8–10 somite pairs) were dissected and stained for hemogenic endothelium marker Runx1, endothelial marker PECAM-1, and AF594-AcLDL. Flow cytometry analysis shows that most Runx1^+^ cells at this stage are AcLDL^+^ (**E**) and PECAM-1 positive (**F**). Scale bar: 20 μm. All values are mean ± SEM. ***p < 0.001; Two-tailed Student’s t-test.

We next sought to further characterize the presence of endothelial and hematopoietic markers in EMPs by immunostaining. 100% of CSF1R^+^ cells express c-kit at E9.0 (15 somite pairs) as we had previously found (Fig. 3A, white arrowheads)^2^, and therefore represent EMPs *at this stage*. Previous reports have shown that EMPs continue to express CSF1R when they differentiate into myeloid cells but lose c-kit expression^2^,^17^,^20^. All stainings were performed in littermates. A significant number of CSF1R^+^ cells also co-express the hematopoietic/endothelial marker CD34 (Fig. 3B, white arrowheads). We found that CSF1R^+^ cells co-express VE-Cadherin and CD31 (data not shown). Interestingly, we observe that the uptake of fluorescently-tagged AcLDL (AF488-AcLDL) labeled endothelial cells but never labeled CSF1R^+^ cells (Fig. 3C). CSF1R^+^ cells were rounded and located away from the vessel walls (Fig. 3C).

AcLDL uptake is mediated by scavenger receptors^36^ and labels differentiated endothelial and macrophages in the adult. Primitive macrophages do not express scavenger receptors^37^ and therefore would not be labeled by AcLDL. We further investigated whether AcLDL could differentially label endothelial cells, EMPs and/or EMP-derived cells. *Csf1r*^*MeriCreMer*^ *Rosa26*^*tdT*^ embryos were dissected at E8.0 (4–6 somite pairs) or E8.5 (10–12 somite pairs), injected intra-vascularly with AF488-AcLDL, and placed in culture. Hydroxy-tamoxifen was added directly to the culture media, and embryos were not exposed to tamoxifen *in utero*. This allowed precise timing of the onset of tdT expression. After 24 hours of culture, embryos were analyzed by flow cytometry. Remarkably, if the embryos were injected before the onset of EMP differentiation, the majority of tdT^+^ cells were also AcLDL labelled (Fig. 3D, 83.9% ±3.2, n = 8). However, if AcLDL was injected just 9 hours later (10–12 somite pairs), at a stage when most tdT^+^ cells are rounded and in circulation (Fig. 1), then only a third of tdT^+^ cells were AcLDL labelled (32.3% ±8.2, n = 4).

To reconcile the differences in our results at E8.0, E8.5 and E9.0, we next used an earlier marker for EMPs, Runx1^2^, to test whether Runx1^+^ cells could also uptake AcLDL. Yolk sacs of embryos at E8.5 (8–10 somite pairs) were dissociated and stained with antibodies against Runx1, PECAM-1, and with AcLDL. We found that 76.4 ± 5.7% of PECAM-1^+^ endothelial cells co-stain with AcLDL (n = 4); and that 84.4 ±4.0% of Runx1^+^ cells are labelled by AcLDL (n = 4, Fig. 3E). Furthermore, 87.3 ±10.6% of Runx1^+^ yolk sac cells were also PECAM-1^+^ (n = 4, Fig. 3F). As such, EMPs are capable of taking up AcLDL while displaying an endothelial-like phenotype, but EMPs and EMP-derived cells in circulation lose this ability.

**Figure 4:**
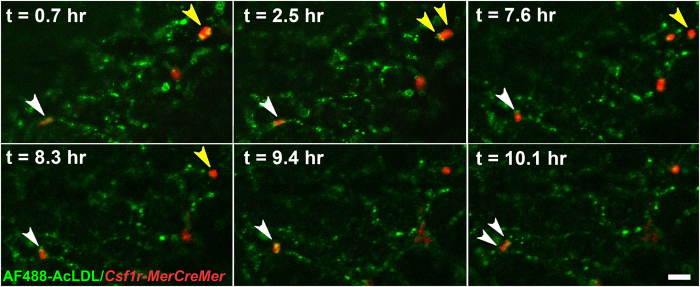
EMPs bud from endothelium and divide prior to entering circulation. *Csf1r*^*MeriCreMer*^*Rosa26*^*tdT*^ embryos were pulsed with tamoxifen *in utero* at E7.75, harvested at E8.5 and injected with Alexafluor488 acetylated low-density lipoprotein (AF488-AcLDL) to label endothelial cells (green). Embryos were cultured for 16 hours in the presence of 4-hydroxytamoxifen and then time-lapsed. Images were taken every 6 minutes on an environment-controlled confocal microscope for 10 hours (see Supplemental Movie 1). A tdT^+^ cell (white arrowhead) appears with an endothelial phenotype in the vessel wall, then rounds and buds up from the endothelium. In the last few frames, this cell begins to divide (t = 10.1 hr, white arrowheads). A second tdT^+^ cell (yellow arrowheads) divides into two cells, and one of the daughter cells enters into circulation (yellow arrowheads, at approximately 7.6 hr). Scale bar: 50 μm.

We took advantage of selective AcLDL uptake to perform time-lapse microscopy of EMP extravasation. Embryos were injected with fluorescent AcLDL at 4–6 somite pairs and then cultured. At E9.0, embryos were transferred to a heated microscope stage. At the start of the time-lapse experiment (Supplemental Movie 1), we identified a tdT^+^ cell that was elongated and integrated in the endothelium and that had been labeled with AcLDL at E8.5 (Fig. 4 snapshots, t = 0.7 hr, white arrowhead). The cell rounded up and budded into the vessel lumen (t = 7.6 to 9.4 hr, white arrowhead). By the end of the time-lapse experiment, the cell began to divide (t = 10.1 hr, white arrowheads). A second tdT^+^ cell can be observed rounded at the beginning of the experiment (Fig. 4, t = 0.7 hr, yellow arrowheads), which divides to form two daughter cells (t = 2.5 to 7.6 hr, yellow arrowheads). One of the daughter cells enters circulation, whereas the other remains in the vessel wall (t = 8.3 hr, yellow arrowhead). We previously observed endothelial cell division frequently preceding cell entry into circulation in avian embryos^38^. Our results here clearly demonstrate that the cells that differentiate into EMPs are luminal and take up AcLDL before EMP commitment occurs but stop taking up AcLDL once they enter circulation. The results also suggest that cell division may be associated with the transition into circulation.

### Blood flow facilitates EMP emergence from the endothelium but is not required for differentiation

**Figure 5:**
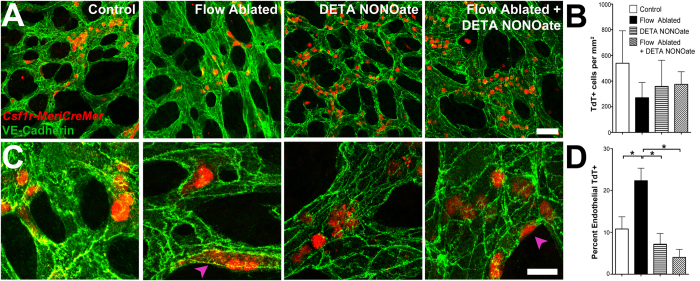
Blood flow facilitates the entry into circulation of endothelial-like EMPs in a nitric oxide-dependent manner. *Csf1r*^*MeriCreMer*^*Rosa26*^*tdT*^ embryos were dissected at 4–5 somite pairs and cultured with normal circulation (n = 5), with ablated circulation (n = 7), in the presence of DETA NONOate (n = 4), and with both DETA NONOate and flow-ablation (n = 4). Yolk sacs were counterstained for VE-Cadherin (**A**, green). Only cells in the plexus proximal to the embryo were analyzed. Quantification confirms that no significant difference in total number of tdT^+^ cells is present under any of the conditions (**B**). Higher magnification images show an increase in endothelial-like tdT^+^ cells in the absence of flow (**C**, purple arrowheads). Flow ablation increases the percent of endothelial-like tdT^+^ cells (**D**). In the presence of exogenous nitric oxide, no difference in the percentage of endothelial-like tdT^+^ cells is observed between control and ablated flow embryos. Scale bars: 50 μm (**A**); 200 μm (**C**). All values are mean ± SEM. *p < 0.05; ANOVA followed by Tukey’s multiple comparisons test.

Since we observed that EMPs first appear around the stage when circulation begins, we investigated whether EMP production and transition from the endothelium into circulation was dependent on blood flow. Previous studies using a genetic mouse model that lacks blood flow reported no difference in the number or morphology of EMPs at E10.0^20^. We surgically ablated blood flow in *Csf1r*^*MeriCreMer*^*Rosa26*^*tdT*^ embryos, using a technique that we previously developed, whereby the inlet to the heart is severed and embryos are cultured overnight^39^. Only viable embryos with a beating heart but no circulating cells were analyzed. In agreement with previous reports^20^, our quantification showed that arresting circulation did not alter the total number of tdT^+^ cells (Fig. 5A,B). With normal circulation, tdT^+^ cells were distributed equally in the vascular plexus, whereas in the absence of flow, EMPs were mostly confined to the blood islands (Supplemental Fig. 6). The majority of tdT^+^ cells were rounded and in circulation in control embryos, but in flow-ablated embryos, a greater number of tdT^+^ cells remained in the endothelium (pink arrowhead, Fig. 5C,D). Only cells in the plexus proximal to the embryo were analyzed.

Nitric oxide production in response to shear stress has been found to decrease adhesion molecules in endothelial cells^40^,^41^ and increase the number of HSCs released by the hemogenic endothelium in zebrafish and cultured embryoid bodies^31^,^32^. We therefore tested whether nitric oxide affected EMP development by treating embryos with a nitric oxide donor (DETA NONOate), in the presence and absence of flow. Treatment with DETA NONOate did not affect the number of tdT^+^ cells (Fig. 5A,B). Flow ablation in embryos, however, had no effect on the transition from the endothelium into circulation when DETA NONOate was present (Fig. 5C,D). Collectively, this shows that blood flow facilitates the transition of EMPs from the endothelium into circulation, but that exogenous nitric oxide alone is sufficient to induce this process. Neither blood flow nor nitric oxide treatment significantly altered the total number of EMPs or EMP-derived cells produced in the yolk sac.

### Early myeloid cells circulate and patrol similarly to adult monocytes

**Figure 6:**
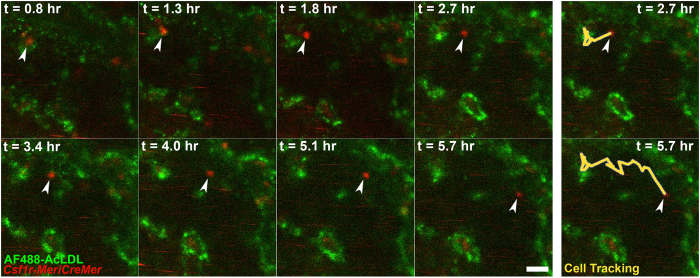
EMP-derived cells circulate but also patrol along the vessel wall. *Csf1r*^*MeriCreMer*^*Rosa26*^*tdT*^ embryos were pulsed with tamoxifen *in utero* at E7.75, harvested at E9.0 and injected with Alexa Fluor488-conjugated acetylated low-density lipoprotein (AF488-AcLDL) to label endothelial cells (green). Images were taken every 6 minutes. An EMP-derived cell (red) intravasates through the vessel wall (green) and enters circulation (white arrowhead). The cell is observed moving within the vessels at speeds much lower than surrounding circulating cells, which can be detected as red streaks. Panels on right hand side indicate cell tracking at 2.7 and 5.7 hours in yellow. Scale bar: 25 μm.

To understand myeloid cell behavior and function during remodeling, we returned to *in vivo* time-lapse microscopy of *Csf1r*^*MeriCreMer*^*Rosa26*^*tdT*^ embryos. Similar to monocytes in adult vasculature, we found that EMP-derived cells were able to move along embryonic vasculature (Supplemental Movie 2, 3). Circulating tdT^+^ cells can be identified by streaks of red moving at high speed in the vessel in these movies. A tdT^+^ cell is first observed perivascularly (Supplemental Movie 2, Fig. 6, t = 0.8 hr), then it enters the circulation and moves within the vessel at speeds much lower than other nearby circulating cells (t = 1.8 to 5.7 hr). Patrolling monocytes in the adult move along the endothelium independent of the direction of flow^42^. The patrolling behavior of EMP-derived cells suggests that they are responsive to signaling molecules on the embryonic endothelium.

### CSF1-Fc injection increases the number of myeloid cells and disrupts venous remodeling

**Figure 7:**
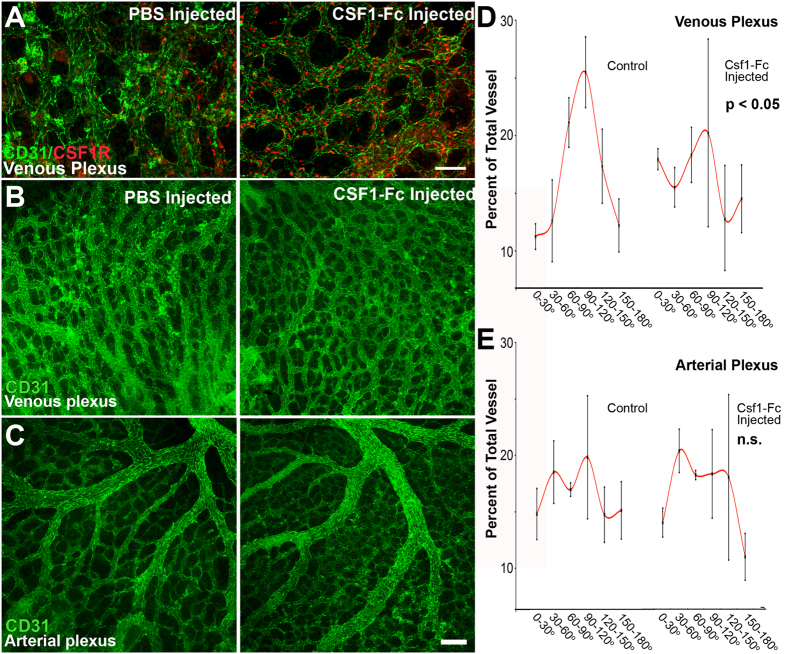
Exogenous CSF1-Fc administration increases the number of early myeloid cells and disrupts remodeling in the venous plexus. Embryos were dissected at E8.5 (10 somite pairs) and injected with either PBS or CSF1-Fc and then cultured for 24 hours. CSF1-Fc treatment resulted in an increase in the number of CSF1R^+^ cells (red) localizing to the venous plexus (**A**, n = 5–6 embryos). Injection of CSF1-Fc disrupted venous (**B**), but not arterial remodeling (**C**). Blinded quantification of vessel angles confirms that remodeling was significantly impaired in the venous plexus (**D**), but not arterial plexus (**E**). Scale bars: 100 μm (**A**), 200 μm (**B**,**C**). All values are mean ± SEM. Distributions were compared using a Kolmogorov-Smirnov test.

The temporal emergence of yolk sac EMPs just at the onset of circulation prompted us to consider if early myeloid cells have a function during vascular remodeling. No vascular defects have been observed in CSF1R loss-of-function studies^5^; therefore, we investigated the effects of CSF1R gain-of-function on vascular development. The cytokine macrophage colony-stimulating factor (M-CSF, or CSF1) can induce the survival, proliferation and differentiation of myeloid cells via interactions with its receptor CSF1R *in vitro* and *in vivo*^43^. We injected wild-type embryos with CSF1-Fc cytokine or PBS at E8.5^43^, cultured embryos for 24 hours, and then immunostained for CSF1R and CD31 (Fig. 7A). At E8.5, all CSF1R^+^ cells are c-kit positive and represent EMPs^2^. Exogenous CSF1-Fc resulted in a striking increase in the number of CSF1R^+^ cells (red cells) compared to control embryos. While we observed that CSF1R^+^ cells are normally equally distributed in the venous and arterial plexus, CSF1-Fc injection caused the vast majority of these cells to localize to the venous plexus. Arterial and venous regions were identified as shown in Supplemental Fig. 7. In normal vasculogenesis, the morphology of the venous plexus begins as a honeycomb shape, and then remodels upon circulation to form parallel vessels that are connected by transverse vessels (Fig. 7B, left and Supplemental Fig. 7). When embryos were injected with CSF1-Fc, the honeycomb patterning was retained, indicating that remodeling of the venous plexus failed to occur (Fig. 7B, right). No effects were observed in remodeling of the arterial plexus (Fig. 7C). We quantified differences in the venous (Fig. 7D) and arterial remodeling (Fig. 7E) by measuring the angle of vessels relative to mean angle for the region (see Methods). Normal venous remodeling results in a Gaussian distribution of angles since the majority of vessels align parallel to one another (Fig. 7D). The distribution of vessel angles of CSF1-Fc injected yolk sacs was randomized, resulting in a relatively flat distribution (Fig. 7D). In the arterial plexus, large arteries and arterioles are separated by capillaries that do not align with the larger vessels. These capillary vessels are much more numerous than the large vessels and dominate the quantification. As such, a Gaussian distribution is not observed in either the control or injected embryos (Fig. 7E). We also investigated other features of vascular morphology, including average vessel diameter, density of branch points, average segment length and the density of avascular regions. These metrics revealed no differences in the arterial and venous plexuses between control and CSF1-Fc injected embryos (Supplemental Fig. 8).

## Discussion

In mammalian^2^ and zebrafish embryos^1^,^2^, myeloid cells first appear just prior to the onset of blood circulation and vascular remodeling, suggesting an important developmental function of these cells in vascular remodeling. This is the first report to show a functional role for EMP-derived cells in developmental vascular remodeling. While macrophages were classically believed to develop from progenitors in the hematopoietic blood islands^34^, the recent finding that EMPs express most endothelial markers and co-reside in the endothelial lining^20^ have led to a general acceptance of an endothelial cell origin to EMPs^16^,^17^. Yet prior to this report, direct evidence for this was lacking. Our findings definitively show that EMPs arise from two non-exclusive extra-embryonic sites: blood hematopoietic islands and endothelial cells of the yolk sac capillary plexus.

While most EMPs do develop in the hematopoietic blood islands, one third of EMPs arise outside this region. We consistently quantified the same percentage of EMPs of non-blood island origin (32%), using two independent quantification techniques: immunostaining (Fig. 1I) and surgical removal (Fig. 2D). In agreement with previous reports^17^,^20^,^21^, we find that regardless of origin, all EMPs co-express genes typical of an endothelial cell profile, such as VE-Cadherin. This therefore raises the possibility that blood island-derived EMPs also arise from endothelial cells in that region of the yolk sac. This is supported by the fact that injection of AcLDL at 4–6 somite pairs leads to labelling of almost all EMP-derived cells. Blood island-derived EMPs differ morphologically from EMPs arising outside the blood islands, displaying a rounded appearance even when they first differentiate at E8.5 (Supplemental Fig. 5). This may represent inherent cellular differences in EMPs with respect to site of origin, or represent extrinsic differences in the biological signals that are present in the densely-packed stationary blood islands, versus the sparsely populated, but dynamic and flow-exposed blood vessels in regions more proximal to the embryo.

We have identified AcLDL uptake as a tool to distinguish endothelial cells from late EMPs. AcLDL has previously been used to stain endothelial cells during development of HSCs from the AGM^10^. We never observed double-labeled cells when we injected *Csf1r*^*MeriCreMer*^*Rosa26*^*tdT*^ embryos with AcLDL at later stages (E9.0). We could only achieve double-labeling if we injected AcLDL at E8.0 and then cultured the embryos for 24 hours. Because we inject the AcLDL intravascularly, we can state that the cells that differentiate into EMPs were in a luminal location at the time of AcLDL injection. The fact that the cells that differentiate into EMPs were able to take up AcLDL indicates that these cells were functionally endothelial cells at the time of injection. The co-expression of Runx1^+^ in AcLDL^+^ endothelial cells at 10 somite pairs (Fig. 3E) does, however, suggest a hemogenic potential at this stage.

Time-lapse analyses of mouse AGM explants have shown that HSCs start budding from the vessel wall but never dissociate completely^11^, perhaps due to the lack of blood flow in the explant model used. In our time-lapse movies where blood flow is ongoing, the EMPs can be seen rounding up from the endothelium, then suddenly disappearing from the region of interest within a single frame: a fast movement that can only be caused by the cell entering blood flow. In this same time-lapse experiment (Supplemental Movie 1), we also observed that only one of the two daughter cells was released into circulation. We could not extend the movie long enough to verify the fate of the second cell. It may have entered circulation or may have reintegrated into the endothelium. Asymmetric cell division, defined as one daughter cell retaining stem-like properties while the other differentiates, is prevalent during stem cell proliferation^44^. Therefore, the difference in the behavior of the two daughter cells is of significant interest.

We found that blood flow is important to stimulate the emergence of EMP-derived cells from the yolk sac endothelium, but does not affect the total number of EMP-derived cells in the vascular plexus. This is somewhat contrary to previous findings, which either show that the number of HSCs produced by the endothelium is increased by shear stress^31^,^32^, or show that neither number nor morphology of EMPs is affected by altered blood flow patterns^20^,^31^,^32^. It is important to note that the Frame *et al*. study used a different model (i.e., *Ncx*^−/−^ embryos), examined a later stage of development (E10.0) and did not distinguish between blood islands and vascular plexus-derived EMPs. Therefore, a number of reasons could explain differences in our results. First, we limited our analyses to the yolk sac vascular plexus more proximal to the embryo, since the large majority of blood island-derived EMPs had a rounded phenotype and would obscure any detectable differences between control and flow-ablated embryos. We believe that this is the principal reason for the difference between our results. *Ncx*^−/−^ embryos are resorbed by E10.5^45^, and may consequently release reactive oxygen as cells die, which could trigger the same events as flow-induced nitric oxide production. Thus, the differences in stage could also explain the discrepancy in findings. Another explanation is that differences are the result of an embryo culture model versus an *in vivo* model. Flow produces nitric oxide, and our results indicate that nitric oxide alone can induce the transition of EMPs into circulation. The fact that we can rescue the flow-abated phenotype using a nitric oxide donor suggests that the observed differences are not simply an artifact of embryo culture. Nitric oxide reduces cell-cell adhesion molecules in endothelial cells^40^,^41^, which would facilitate the transition from the endothelial phenotype to the rounded phenotype.

In order to investigate the function of early myeloid cells in vascular remodeling, we injected embryos with exogenous CSF1-Fc ligand, which resulted in an increase in CSF1R^+^ cells that localized primarily to the venous plexus. We did not observe a difference in the arterial-venous identity of endothelial cells that produce EMPs at E8.5 (data not shown), as others have also reported^20^. We also randomized injection location such that increase in venous myeloid cells cannot be explained by limited dispersal of CSF1-Fc. In adults, leukocytes only extravasate in post-capillary venules, which is thought to occur due to the lower shear forces present in venules^46^. Thus, the venous positioning of CSF1R^+^ cells when stimulated with CSF1-Fc may have resulted from increased adherence and/or extravasation in veins compared to arteries, rather than an increase in myeloid cell production by venous endothelial cells. The observed defects being limited to the venous plexus may have also been due to the location of CSF1R^+^ cells rather than a venous-specific activity of these cells. Other reports have previously shown that in the adult, ablation of CSF1 results in reduced vascular density and remodeling^28^, whereas increased CSF1 enhances macrophage recruitment, angiogenesis and remodeling in tumor tissues^47^. Similarly, we find that in the embryo, CSF1/CSF1R signaling must be tightly regulated during vascular remodeling; as exogenous CSF1-Fc disrupts venous remodeling.

Collectively, this is the first report that conclusively delineates a functional and spatiotemporal relationship of yolk sac EMPs with yolk sac endothelial cells. We demonstrate that EMPs can arise from the vascular endothelium *in vivo* in a flow-dependent, nitric oxide-induced mechanism, that they behave and patrol in embryonic blood vessels similar to adult monocytes, and lastly show that early myeloid cells are important for early vascular remodeling.

## Experimental Procedures

### Transgenic mouse models

*Rosa26*^*CAG-LSL-tdTomato*^ Cre-reporter males (JAX # 007909) were mated with either constitutive *Csf1r*^*iCre*^ (JAX # 021024) or inducible *Csf1r*^*MeriCreMer*^ (JAX # 019098) females. Unless otherwise stated, inducible *Csf1r*^*MeriCreMer*^ dams were injected at E7.75 with a single intraperitoneal dose of tamoxifen dissolved in corn oil (75 mg/kg of body weight, Cayman Technologies, # 13258). Animal use procedures conformed to the guidelines of, and were approved by, the Ethics Committees for Animal Use of KU Leuven and McGill University Health Centre.

### Antibodies

The following antibodies were used for flow cytometry and immunohistochemistry analysis: CD31 (BD Bioscience, #553371; DSHB, 2H8), CD34-FITC (eBiosciences, #11-0341), Ter119-PE (BD Biosciences, #553673), Runx1 (Abcam, ab92336), c-kit-FITC (eBiosciences, #11-1171), CSF1R (Abcam, #Ab32633; eBioscience AFS98), and VE-Cadherin (R&D, #AF1002). Alexa-Fluor tagged secondary antibodies (Invitrogen) were used for all non-conjugated primary antibodies with the exception of anti-Armenian Hamster secondary (AlexaFluor647, Abcam).

### Embryo culture and manipulation

To isolate the origins of EMPs, hematopoietic blood islands were dissected from embryos at 3–5 somite pairs using #55 forceps. To impede blood flow and shear stress, the vitelline vein was severed pre-circulation (4–5 somite pairs) using #55 forceps to prevent cardiac output flow^39^,^48^. To mimic the release of nitric oxide induced by shear stress in cultured embryos, media was supplemented with 10 μM of DETA NONOate. In all experiments, embryos were placed in culture and allowed to develop *ex vivo* for 16–24 hours, as previously described^33^. Only data from embryos with a visibly beating heart after culture were used for analysis. For all culture experiments with the inducible Cre model, 10 μM 4-hydroxytamoxifen (Sigma, # H6278) was added to the culture media.

### Flow cytometry processing and analysis

Embryos were harvested and dissected at the stage indicated in 20% FBS in PBS. Care was taken to remove the Reichert’s membrane and ectoplacental cone, which have CSF1R-expressing syncytiotrophoblasts. Isolated yolk sacs were dissociated from the embryo proper, placed in separate tubes, then processed and analyzed as described^49^, and according to antibody manufacturer instructions. 7-AAD or EF780 viability dye was used to exclude dead cells and debris. Flow cytometry was performed using FACSCanto II or FACS AriaIII and data analysis was performed using FACS Diva software and Flow Jo. Gating controls were performed with yolk sacs that were non-stained, single stained and double-stained, and, when appropriate, secondary antibody only stained (Supplemental Fig. 3).

### Quantification of vascular morphology

Images of the vasculature were thresholded and skeletonized to identify all branch points. The angle of all vessels was measured in Photoshop. The average angle for all vessels was subtracted from each angle measurement, then 90 degrees was added to normalize the orientation of each image. The distribution of angles between treatments was analyzed using a Kolmogorov-Smirnov test. Other vessel morphology characteristics were analyzed from the thresholded images using Biologic Analyzer software.

## Additional Information

**How to cite this article:** Kasaai, B. *et al*. Erythro-myeloid progenitors can differentiate from endothelial cells and modulate embryonic vascular remodeling. *Sci. Rep.* **7**, 43817; doi: 10.1038/srep43817 (2017).

**Publisher's note:** Springer Nature remains neutral with regard to jurisdictional claims in published maps and institutional affiliations.

## Supplementary Material

Supplemental Information

Supplementary Movie S1

Supplementary Movie S2

Supplementary Movie S3
