# Supplementary material for: Erythro-myeloid progenitors can differentiate from endothelial cells and modulate embryonic vascular remodeling
Source: Sci Rep. 2017 Mar 8;7:43817. doi: 10.1038/srep43817 (PMC5341067; doi:10.1038/srep43817)
Supplement: Supplemental Information [file srep43817-s1.pdf]

Erythro-myeloid progenitors can differentiate from endothelial cells and modulate embryonic vascular remodeling

SUPPLEMENTAL DATA

Bahar Kasaai[1,2], Vincenza Caolo[1], Hanna M. Peacock[1], Stephanie Lehoux[3], Elisa Gomez Perdiguero[4], Aernout Luttun[1], Elizabeth A.V. Jones[1*]

[1]Department of Cardiovascular Sciences, Centre for Molecular and Vascular Biology, KU Leuven, Herestraat 49 - box 911, 3000 Leuven, Belgium

[2]Institute of Human Genetics, CNRS, 141 rue de la Cardonille, 34396, Montpellier, France.

[3]Lady Davis Institute, Department of Experimental Medicine, McGill University, 3755 Ch. Côte-Ste-Catherine, Montréal, QC, H3T 1E2, Canada

[4]CNRS URM 3738, Institut Pasteur, 25-28 Rue du Docteur Roux, Paris 75015, France

SUPPLEMENTAL MATERIAL

Video S1 – Time lapse of Csf1r-derived cell division and entry into embryonic circulation.

Csf1r^{MeriCreMer}*Rosa26*^{tdT} embryos were pulsed with tamoxifen at E7.75, harvested at E8.5 and injected with Alexafluor488 conjugated AcLDL to label endothelial cells (green). Embryos were cultured overnight in the presence of 10 μM 4-hydroxytamoxifen and time-lapsed the following morning on an environment-controlled microscope stage. Confocal laser scanning microscope was used to take an image every 6 minutes for a total time (t) of 10 hours. In the middle of the frame, a tdT^{+} cell (white arrow) appears with an endothelial phenotype in the vessel wall, then rounds and buds up from the endothelium, and begins to divide (t=10.1 hr). In the top right of the frame, a tdT^{+} cell divides into two cells (yellow arrowheads), and one of the daughter cell enters into circulation. Scale bar: 50μm.

Video S2 – Csf1r-derived cell circulate but also patrol in the vessel wall.

Csf1r^{MeriCreMer}*Rosa26*^{tdT} embryos were pulsed with tamoxifen at E7.75, harvested at E9.0 and injected with Alexafluor488 conjugated AcLDL to label endothelial cells (green). Confocal laser scanning microscope was used to take an image every 6 minutes. An EMP (red) intravasates through the vessel wall (green) and enters circulation (white arrow). The cell is observed moving within the vessels at speeds much lower than surrounding circulating cells that can be detected as red streaks. Scale bar: 25μm.

Video S3 – Csf1r-derived cell patrolling in the vessel wall.

Csf1r^{MeriCreMer}*Rosa26*^{tdT} embryos were pulsed with tamoxifen at E7.75, harvested at E9.0 and injected with Alexafluor488 conjugated AcLDL to label endothelial cells (green). Confocal laser

scanning microscope was used to take an image every 6 minutes. An EMP (red) is observed moving within the vessels. Cells in circulation appear as streaks in single frames due to their high velocity. In the last frames, the EMP disappears, possibly through circulation or by extravasating out of the plane of focus. Scale bar: 25μm.

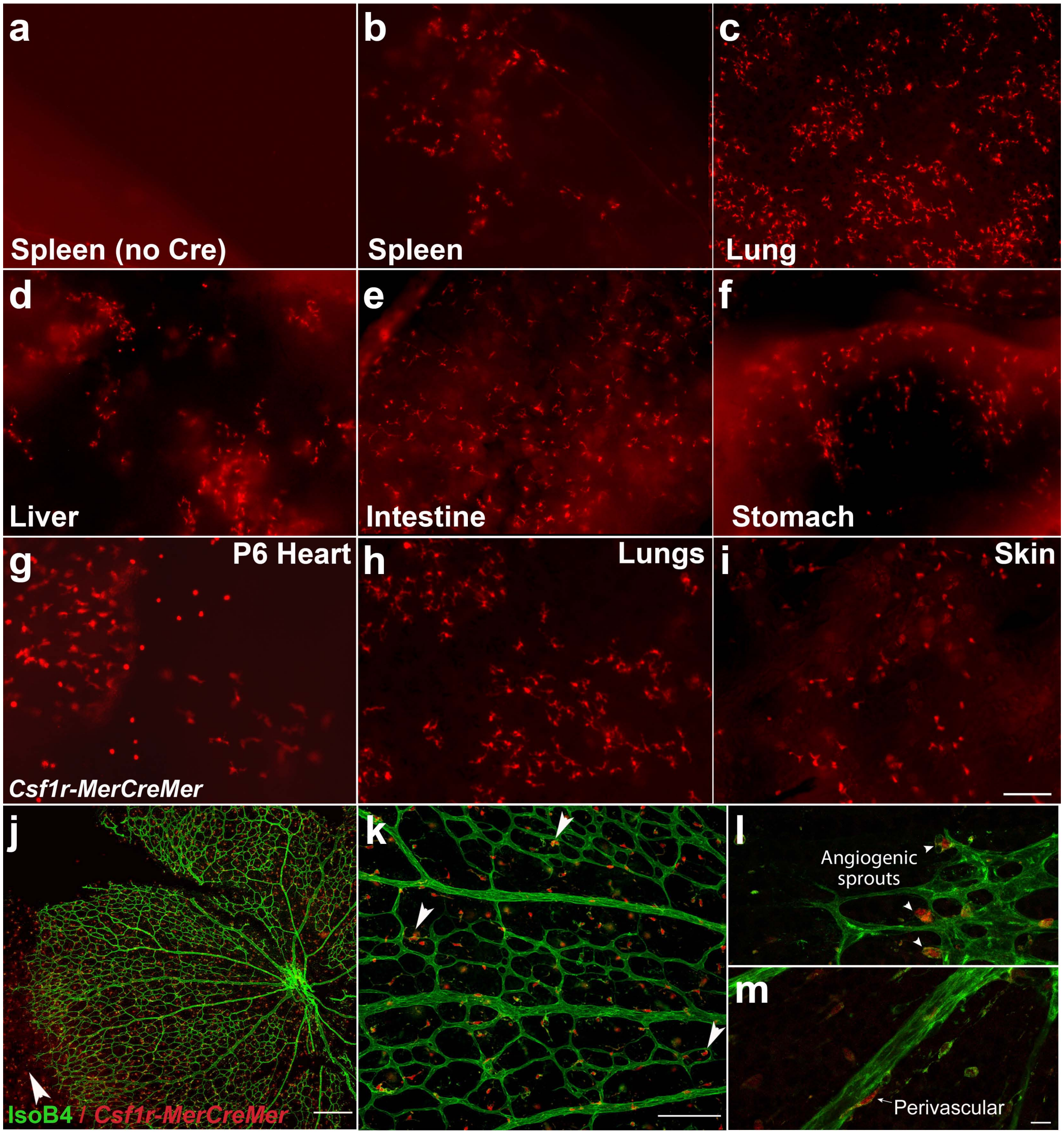

Figure S1 – Yolk sac-derived EMPs contribute to neonatal tissue resident macrophages and vascularizing retinas, where they home to regions of angiogenic sprouts, remodeling and anastomosis. *Csf1r*^{MeriCreMer}*Rosa26*^{tdT} embryos were pulse with tamoxifen at E7.75, such that all yolk sac derived EMPs are labeled with tdTomato throughout fetal and neonatal development. Organs were harvested from 6-day-old pups (P6). Yolk sac-derived tdT^{+} cells (red) were present in all organs harvested including the spleen, lungs, liver, intestine, and retinas (A to M). Developing P6 retinas were counterstained with IsolectinB4 to visualize blood vessels (J-M, green). tdT^{+} cells were widely distributed throughout the retina (D-E), and associated with the radial periphery, a site of continuous angiogenesis (J, white arrow); anastomosing loops (K, arrows) and angiogenic sprouts (L). In the more mature regions of the network, tdT^{+} cells often resided in perivascular locations of the matured vessels (M). Scale bars: 200μm (A-I), 250μm (J), 100μm (K), and 20μm (M).

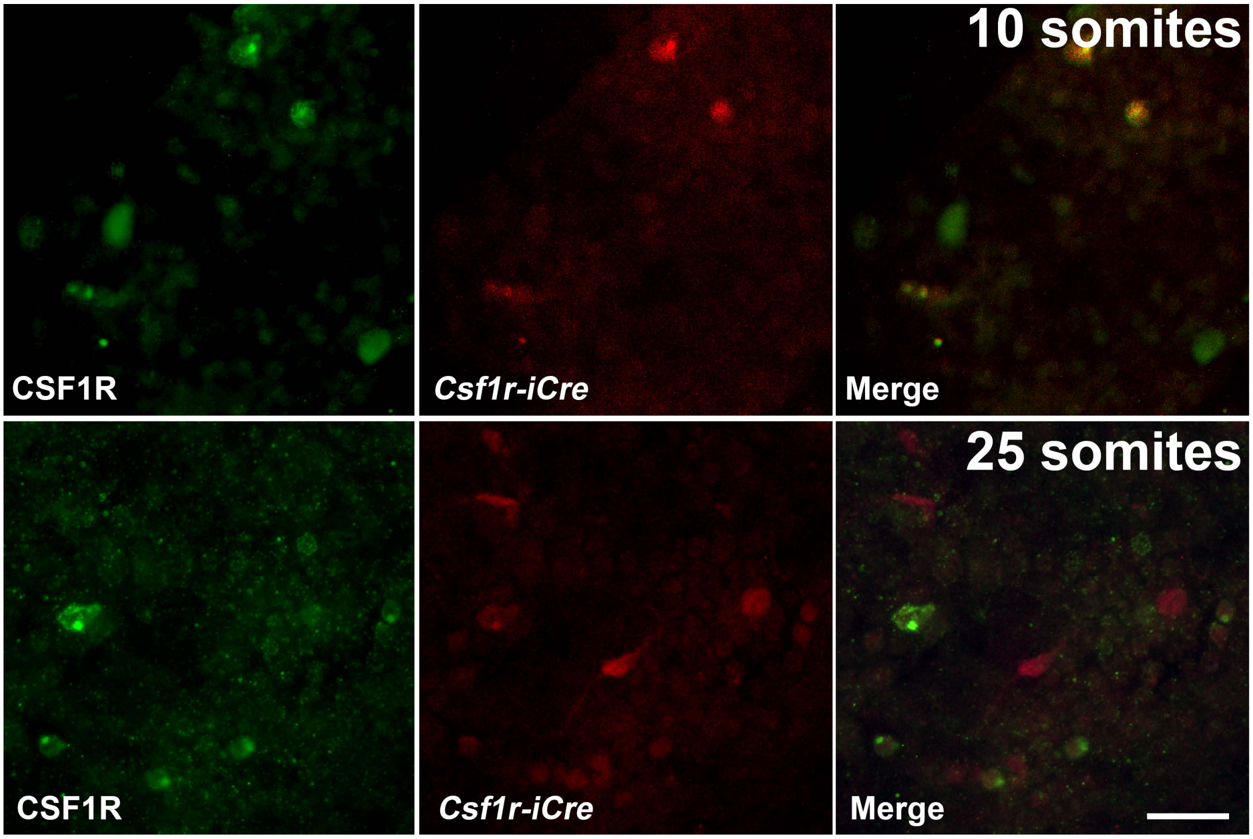

Supplemental Figure 2 – CSF1R expression in tdT-labelled cells. Yolk sacs from *Csf1r*^{iCre}*Rosa26*^{tdT} embryos were collected at E8.5 (10 somites) and at E9.5 (25 somites) and stained for CSF1R protein. (n=3 embryos each). At 10 somites, all tdT+ cells express CSF1R protein. The same is not true at 25 somites, indicating that some EMP-derived cell lose CSF1R expression. Scale bar: 50 μm.

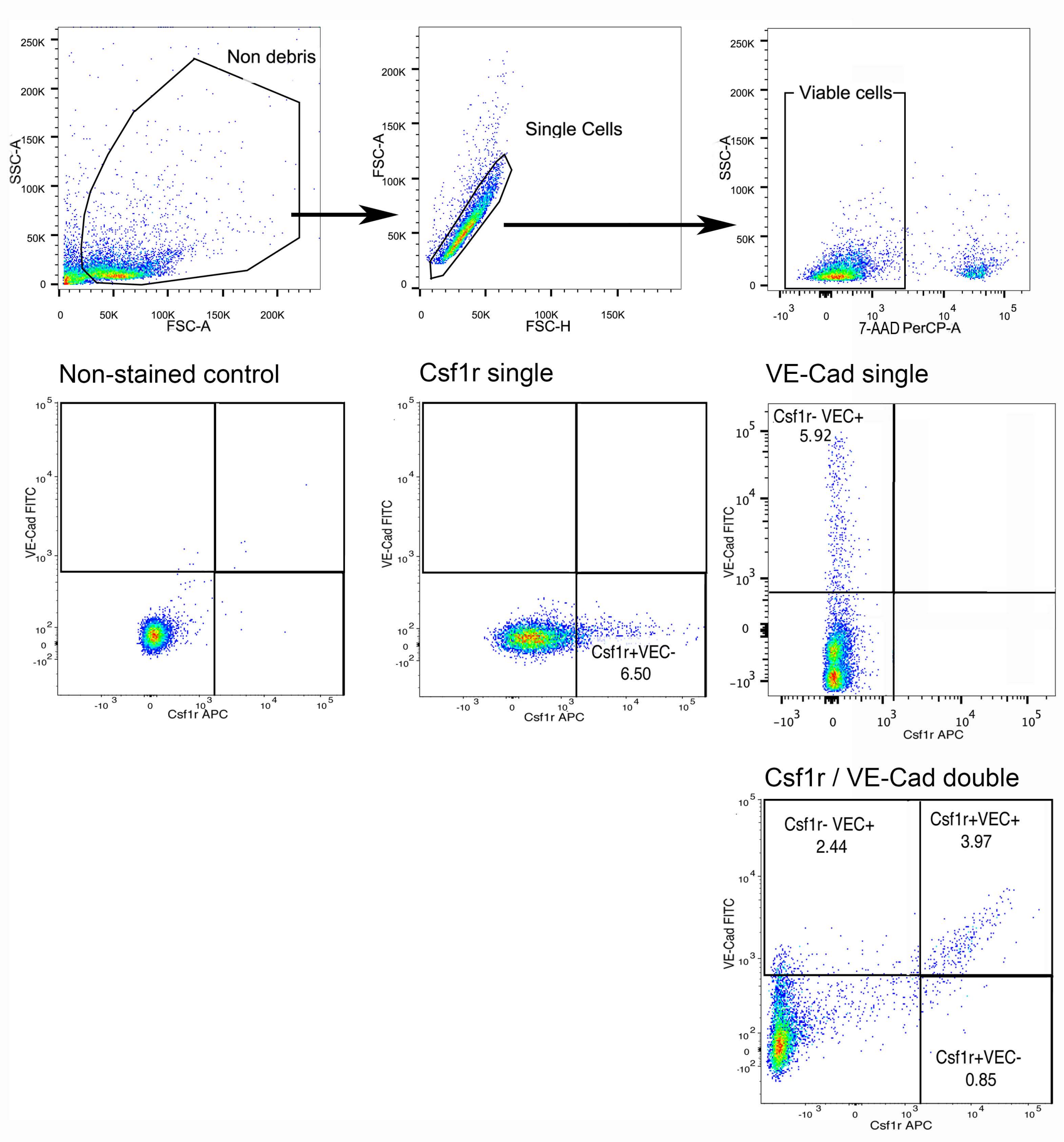

Supplemental Figure 3 – Representative scatter plots of flow cytometry from E8.5 isolated yolk sacs. Yolk sacs were isolated from embryos (in this case, 10 somites), treated with Collagenase (0.5mg/mL) and DNAseI (100μg/mL) for 35 min, mechanically dissociated, blocked and stained with annotated antibodies. Either 7AAD PerCP (shown here) or EF780 viability dye was used to exclude cell debris and non-viable cells. SSC-A vs FSC-A scatterplot is representative of all viable, single cell population derived from E8.5 yolk sacs. All experiments, controls and compensation were performed using either FACS Canto II or FACS AriaIII analyzer. All flow cytometry analyses of yolk sacs were performed in a similar manner.

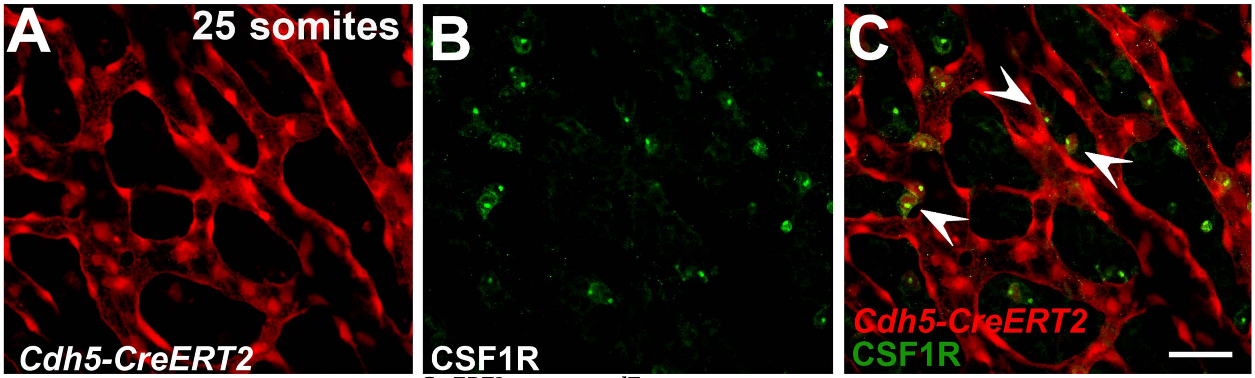

Supplemental Figure 4 – *Cdh5^{CreERT2} Rosa26^{tdT}* labelled cells differentiate into CSF1R+ cells. Yolk sacs from *Cdh5^{CreERT2}Rosa26^{tdT}* embryos injected with tamoxifen at E7.5 and collected at E9.5 (A-C) show that some myeloid cells (B, green) express endothelial cell lineage marker (arrowheads, C, n=3 embryos). Scale bar: 50 μm.

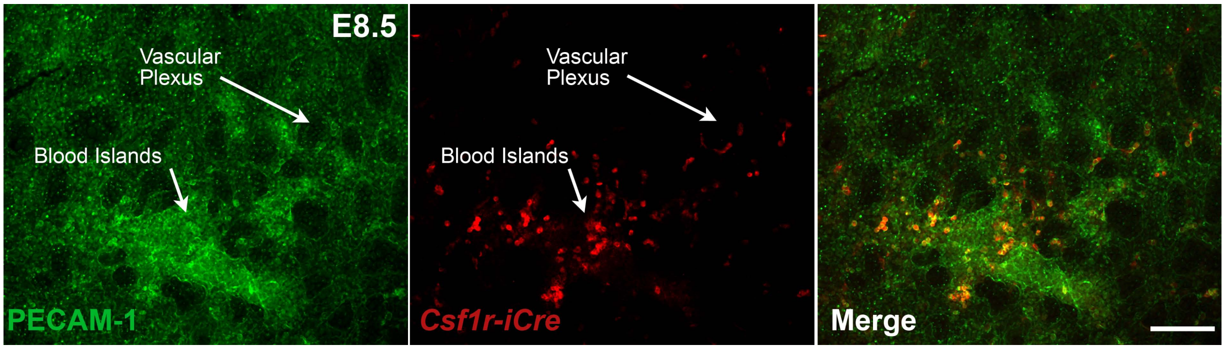

Supplemental Figure 5 – Two phenotypes are present at 10 somites depending on location within the yolk sac. *Csf1r^{iCre}Rosa26^{tdT}* embryos were harvested at E8.5 (10 somites). Yolk sacs were immunostained for PECAM-1. tdT^{+} cells located in the blood islands were for the most part rounded in morphology, whereas tdT^{+} cells that developed in the vascular plexus distal to the blood islands had elongated endothelial morphology and co-localized with the vessel wall. Scale bar: 100 μm.

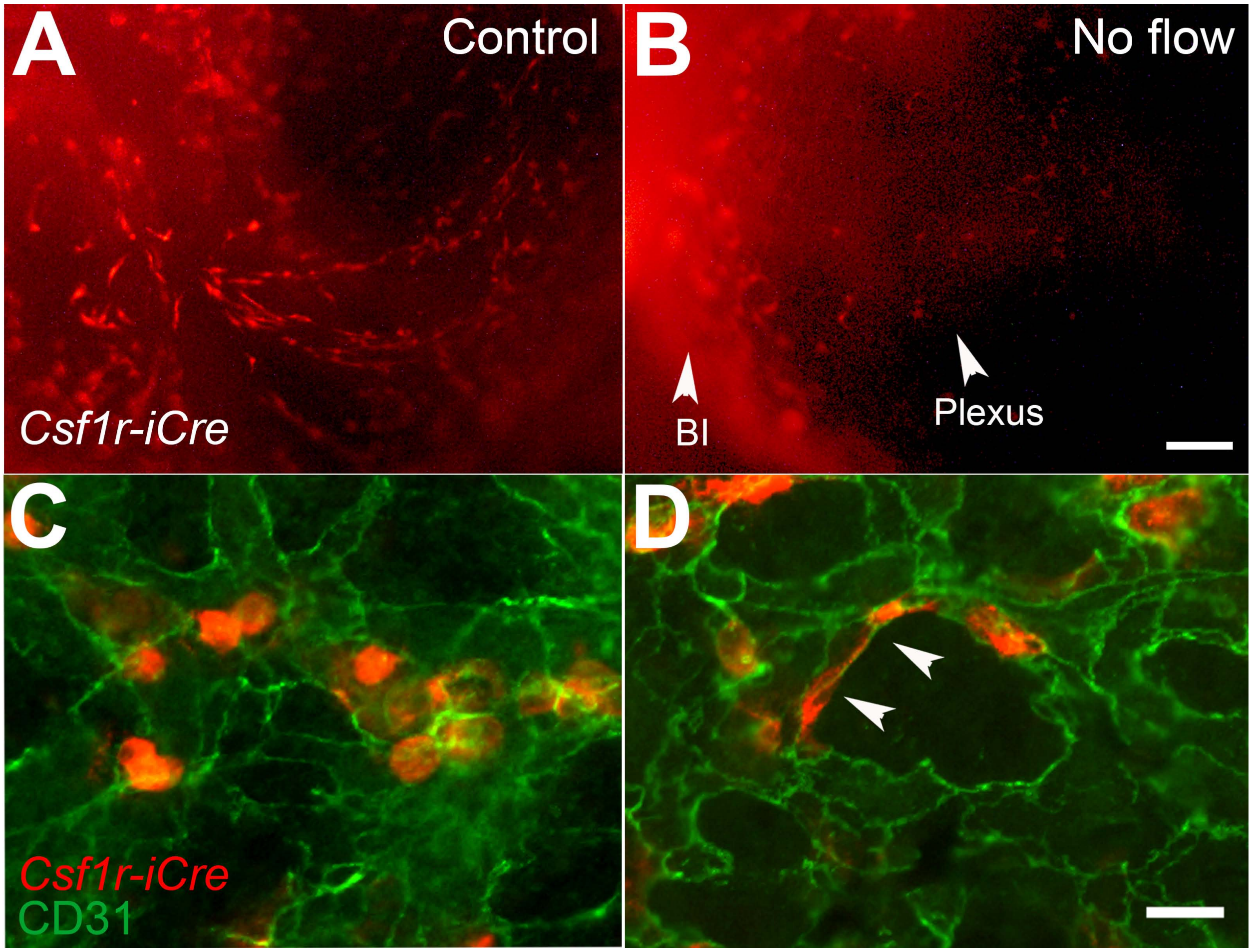

Supplemental Figure 6 – Distribution of CSF1R-derived cells with and without flow. *Csf1r*^{iCre}*Rosa26*^{tdT} embryos were harvested at E8.0 (4-5 somites). Embryos were cultured with (A) and without flow (B). tdT^{+} cells concentrated in the region of the blood islands (BI) in the absence of flow as compared to control (A, n=5; B, n=4). Yolk sacs were immunostained for PECAM-1 (C-D). tdT^{+} cells show elongated endothelial-like cell shapes in the absence of flow (D). Scale bar: 200 μm (A-B), 100 μm (C-D).

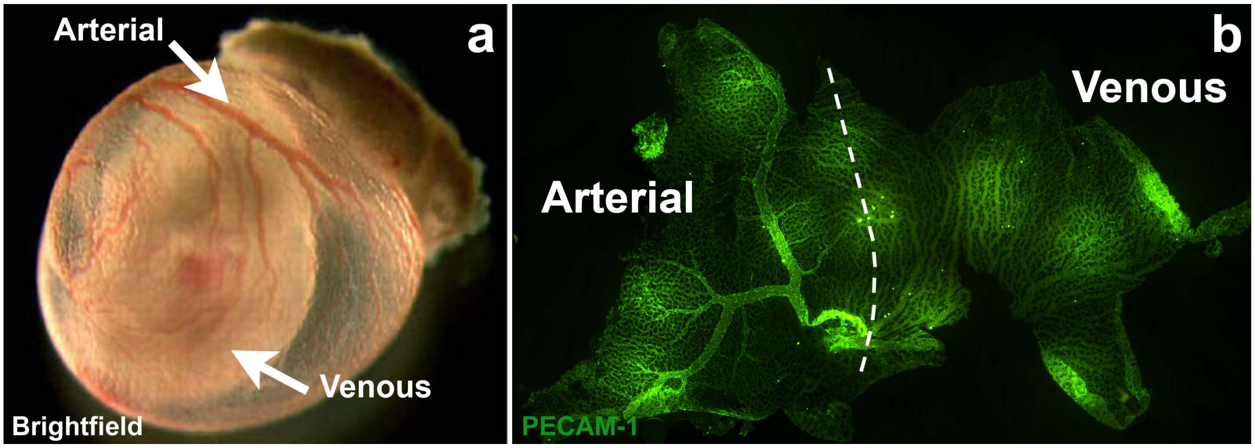

Supplemental Figure 7 – Schematic for identification of arterial/venous regions. In early vascular developmental, a spatial separation of arterial and venous blood vessels is present within the yolk sac. At E9.5, the arterial plexus has large branched vessels that are visible in brightfield images whereas in the venous regions, vessels are smaller (A). The difference can be more easily identified after immunostaining for an endothelial cell marker, in this case PECAM-1 (B). The large arterial vessels are separated by regions of honeycomb-shaped capillaries. The venous vasculature has smaller vessels where the majority of vessels align parallel to one another. The border between the regions is not exact based on these criteria; however, analysis was limited to regions well within either the arterial or venous plexus. Brightfield image taken from [1]. PECAM-1 image previously published in [2].

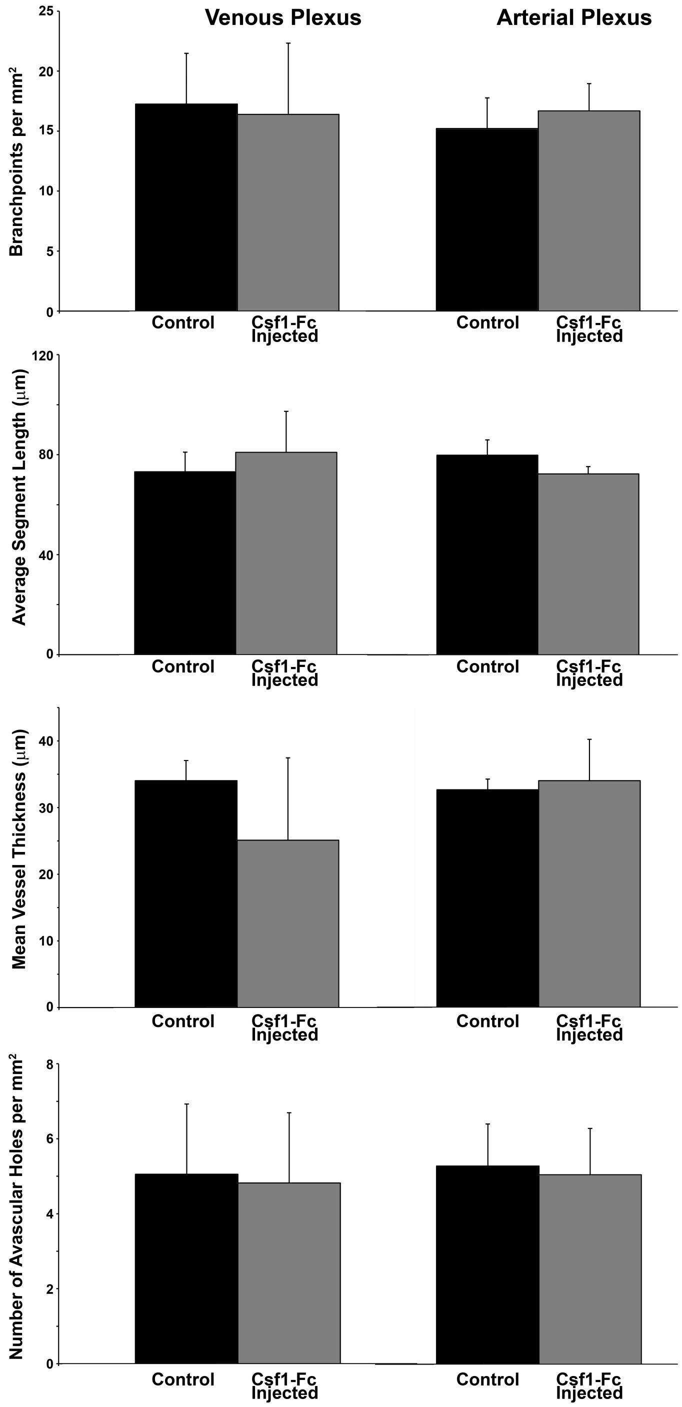

Supplemental Figure 8 – Morphological measurements of arterial and venous plexus after CSF1-Fc injection. Embryos were injected with PBS (control) or CSF1-Fc at E8.5 and cultured for 24 hours. Yolk sacs were then immunostained for CD31 and imaged. The density of branchpoints, average segment length, mean vessel thickness and the density of avascular holes was quantified using Biologic Image Analysis software. Arterial and venous vascular networks were identified based on location within the yolk sac. No differences in these metrics were observed between control and CSF1-Fc injected, or between the arterial and venous plexus.

REFERENCES

1 Astorga, J. & Carlsson, P. Hedgehog induction of murine vasculogenesis is mediated by Foxf1 and Bmp4. *Development* **134**, 3753-3761, doi:10.1242/dev.004432 (2007).

2 Prahst, C. *et al.* The H2.0-like homeobox transcription factor modulates yolk sac vascular remodeling in mouse embryos. *Arterioscler Thromb Vasc Biol* **34**, 1468-1476, doi:10.1161/ATVBAHA.114.303626 (2014).
